# Misidentification of Acute Psychiatric Symptoms in the Emergency Room: Clinical Experience in China

**DOI:** 10.3389/fpsyt.2020.579484

**Published:** 2021-01-14

**Authors:** Fang Liu, Jianjun Chen, Yang Du, Wenxia Jiang, Lei Gong, Jun Mu

**Affiliations:** ^1^Department of Neurology, The First Affiliated Hospital of Chongqing Medical University, Chongqing, China; ^2^Institute of Life Sciences, Chongqing Medical University, Chongqing, China; ^3^Department of Neurology, Suining Central Hospital, Sichuan, China

**Keywords:** acute psychiatric symptoms, misidentification, differential diagnosis, clinical characteristics, emergency room

## Abstract

**Introduction:** Patients who come to the emergency department because of acute psychiatric symptoms are often not admitted to the correct department timely. The purpose of this study is to identify the clinical characteristics of patients with acute psychiatric symptoms in order to achieve early and correct triage in the emergency room.

**Methodology:** We conducted a cross-analysis of inpatients who first came to the emergency department with acute psychiatric symptoms and then admitted to the department of neurology or psychiatry between years 2012 and 2018. Among them, 70 patients were rediagnosed and retransferred, with 38 patients to the department of neurology and 32 patients to the department of psychiatry. The clinical characteristics, laboratory examination, and Neuropsychiatric Inventory (NPI) were analyzed.

**Results:** Patients who were rediagnosed with neurological diseases were more prone to have somatic symptoms (headache, dizziness) (*P* < 0.05). Because of the atypical early clinical manifestations in the emergency room, fever and positive neurological signs do not necessarily represent the diagnosis of neurological diseases. On the other hand, the absence of these manifestations does not guarantee the diagnosis of psychiatric illness. According to NPI, abnormal behaviors, changes in appetite, and sleep disturbances were more obvious in patients with neurological diseases (*P* < 0.05), whereas patients with psychiatric disorders often showed prominent irritability (*P* < 0.05).

**Conclusions:** Acute psychiatric symptoms are usually complex and diverse. The triage and diagnosisshould be based on multiple factors. After triage, clinical symptoms should be dynamically observed.

## Introduction

The number of patients with mental disorders is increasing worldwide. According to a survey in the United States, one in eight emergency room visits involved acute psychiatric symptoms (1, 2). Conditions behind these psychiatric emergencies often include mental and substance use disorders (2), neurological (such as encephalitis, stroke, multiple sclerosis, etc.), endocrine, and metabolic diseases (3). Sometimes the situations can be life-threatening; as a result, it is critical to make judgments in a timely and accurate manner. Thus, the patients can be admitted to the proper department right away. However, identification of an underlying medical condition presenting as acute psychiatric symptoms can be very challenging sometimes, given the broad spectrum of diseases in the field of psychiatry and neurology. It was estimated that the average emergency room duration of stay for patients with psychiatric symptoms was three times longer than those presenting with physical illnesses and injuries (4).

There are several reasons for this. First, because of the complexity of brain structure and function, neurology and psychiatry have been intertwined since the establishment of the discipline. Many diseases even have overlaps in the pathogenesis, clinical manifestations, and treatment options (5). Second, the clinical manifestations of such patients are often atypical. Fever, seizures, and some focal neurological deficits may not present in the early stages of the disease. Besides, the course of illness is usually short. There is no previous mental stress and no family history of mental illness. Third, these patients with psychiatric symptoms often cooperate poorly in the history taking and neurological as well as physical examinations. Fourth, the triage system of the emergency room in China needs to be improved. The nurses, not the doctors, decide whether the psychiatrists or neurologists see the patient first. According to hospital regulations, if a neurologist was referred, the patient must be admitted immediately to the department of neurology, and vice versa. There is no acute medical unit to accommodate these patients. Logically, several important examinations, such as lumbar puncture, electroencephalography (EEG), and head magnetic resonance imaging (MRI) cannot be completed in the emergency room. Consequently, some patients failed to be admitted to the proper department timely.

In order to allow patients to be admitted to the correct department as soon as possible, at present, the best solution is to identify convenient and efficient clinical parameters, which help to improve the accuracy of triage. Most of the studies that can be retrieved are based on the diagnosis of mental illness. Some red flags were suggested to exclude mimics (3). These include abnormal vital signs, significant abnormalities on physical and neurological examination, impaired consciousness, visual hallucinations, and recent-onset incontinence (6–8). There was also some kind of literature discussing the classification and diagnosis of organic mental disorders (9). Only one literature analyzed 947 inpatients who requested a psychiatric consultation. It was found that 14.6% had a final diagnosis of mental disorder. Advanced age was significantly associated with an increased risk of misidentification of psychiatric symptoms (10). No literature, so far, cross-analyzed the characteristics of those inpatients who were misdiagnosed by both psychiatrists and neurologists. Our study was the first to sort out all inpatients with acute psychiatric symptoms in both departments of neurology and psychiatry. Efforts have been made to comprehensively analyze clinical characteristics, laboratory tests, and imaging results to better distinguish these patients promptly.

## Methodology

### Participants

From January 2012 to December 2018, inpatients with acute psychiatric symptoms who first came to the emergency room of the First Affiliated Hospital of Chongqing Medical University and then admitted to the department of neurology or psychiatry were screened and cross-analyzed. Patients were enrolled if they were rediagnosed and retransferred. It should be noted that in the emergency room, as a routine, the emergency nurses will first record patients' vital signs and document the onset time, predisposing factors, medical history, family history, history of medication, and substance abuse, before they triage the patients to psychiatrists or neurologists. They also interview the accompanying family members and close friends, especially when the patients are unable to cooperate with the collection of medical history (11).

### Statistical Analysis

SPSS 26 software package was applied to process the statistic (IBM Corporation released in 2019, version 26.0, for Macintosh). For categorical variables (dichotomous variables), such as gender, clinical symptoms and signs, and laboratory test results, the χ^2^ test was used. There are some principles: (1) all theoretical numbers *T* ≥ 5 and total sample size *n* ≥ 40, tested by Pearson χ^2^; (2) if the theoretical number *T* < 5 but *T* ≥ 1, and *n* ≥ 40, the χ^2^ of the continuity test was used; (3) if there was a theoretical number *T* < 1 or *n* < 40, the Fisher test was used. Continuous variables, such as age, course of illness before admission, transfer time, length of stay in the hospital, and the number of consultations, were examined by the Shapiro–Wilk first. If the data were normally distributed, the Student *t* test was used; if not, the non-parametric Mann–Whitney *U* test was used. *p* < 0.05 was considered statistically significant.

## Results

### Confirmed Diagnosis

**Figure 1:**
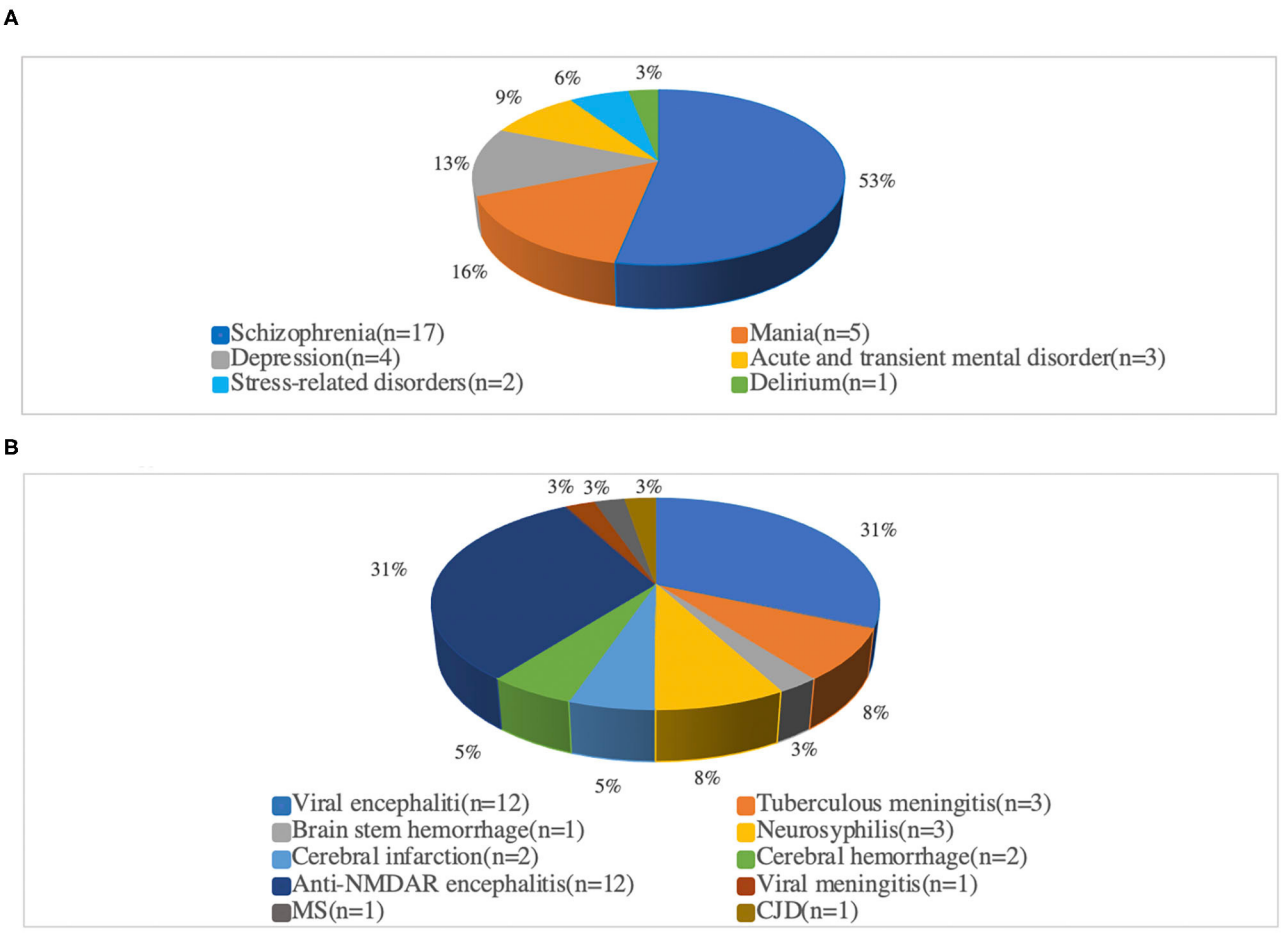
Proportionof each disease after re-diagnosis. **(A)** Admitted to the Department of Neurology, and finally diagnosed with psychiatric related disease. **(B)** Admitted to the Department of psychiatric, and finally diagnosed as neurology related disease. Anti-NMDAR encephalitis, Anti-N-methyl-D-aspartate receptor encephalitis; MS, Multiple sclerosis; CJD, Creutzfeldt-Jakob disease.

A total of 168 patients with acute psychiatric symptoms who first came to the emergency room were sorted out. Ninety-eight patients were correctly triaged and diagnosed. However, after the initial triage, 70 patients had to be retransferred because of an incorrect diagnosis. Among them, 32 patients were transferred from the department of neurology to the department of psychiatry. The most commonly misdiagnosed disease was schizophrenia (17/32, 53.1%). Similarly, 38 patients were transferred from the department of psychiatry to the department of neurology. The most misdiagnosed diseases were anti–*N*-methyl-d-aspartate receptor (NMDAR) encephalitis (12/38, 31.6%) and viral encephalitis (12/38, 31.6%). Figure 1 shows the proportion of each disease.

### Clinical Characteristics

Thirty-seven (52.9%) of the 70 patients were female. The mean age was 34 ± 16.2 years with a range from 12 to 67 years. The clinical characteristics of 70 patients are shown in Table 1. In general, there was no difference in age and gender between the two groups. The somatic symptoms were more commonly seen in neurological diseases (*P* < 0.05). Compared with psychiatric diseases, the duration of neurological diseases and the number of consultations before admission were longer and more frequent (*P* < 0.05). Interestingly, patients with psychiatric disorders had more fever and positive meningeal irritations (*P* < 0.05). In addition, predisposing factors (such as psychological stress, flu history), family history of mental illness, history of physical illness and mental illness, and even Babinski sign were of no significance in distinguishing the diseases (*P* > 0.05).

**Table 1:** Clinical characteristics.

**Baseline characteristics**	**Admitted to DN (*n* = 32)**	**Admitted to DP (*n* = 38)**	***P*-value^†^**
Age (years)	32.9 ± 17.0	34.7 ± 15.8	0.465
Male	18 (56.3%)	15 (39.5%)	0.161
Female	14 (43.7%)	23 (60.5%)	0.161
Admission to transfer time (days)	5.7 ± 4.5	5.9 ± 6.3	0.073
The number of days in hospital	24.0 ± 8.4	22.0 ± 13.8	0.753
The number of consultations	1.7 ± 1.0	2.2 ± 1.2	0.024^*^
Course of illness before admission (days)	6.5 ± 6.3	47.5 ± 123.5	0.002^*^
History of previous physical diseases	8 (25.0%)	9 (23.7%)	0.898
History of previous mental illness	3 (9.4%)	0 (0.0%)	0.091
Family history of mental illness	0 (0.0%)	5 (13.2)	0.058
Predisposing factors	17 (53.1%)	13 (34.2%)	0.111
Fever	13 (40.6%)	0 (0.0%)	<0.001^*^
Somatic symptoms^a^	0 (0.0%)	7 (18.4%)	0.013^*^
Meningeal irritation	4 (12.5%)	0 (0.0%)	0.039^*^
Babinski sign	0 (0.0%)	0 (0.0%)	1.000

*DN, department of neurology; DP, department of psychiatry*.

a *Somatic symptoms include headache and dizziness*.

*Clinical characteristics of 70 patients, the patient data in the table were collected in the emergency room; 32 patients were admitted to the DN; 38 patients were admitted to the DP*.

† *P-values were obtained with the χ^2^ test for categorical variables and the Mann–Whitney U test for continuous variables*.

* *Significance threshold P < 0.05*.

### Laboratory Tests and Imaging Results

The patients initially admitted to the department of neurology or psychiatry had significant statistical differences in the following aspects (*P* < 0.05) (Table 2): (1) blood tests: percentage of neutrophils, and procalcitonin; (2) cerebrospinal fluid examinations: cell count, protein level, virus antibodies, and autoimmune encephalitis–related antibodies; (3) EEG; (4) head MRI.

**Table 2:** Results laboratory tests and imaging results.

**Laboratory anomaly indicators**	**Admitted to DN (*n* = 32)**	**Admitted to DP (*n* = 38)**	***P*-value^#^**
WBC	9 (28.1%)	18 (47.3%)	0.099
Percentage of neutrophils	6 (18.75%)	19 (50.0%)	0.007^*^
PCT	2 (6.2%)	16 (42.1%)	0.002^*^
d-Dimer	4 (12.5%)	6 (15.8%)	0.961
Creatine	1 (3.1%)	2 (5.3%)	1.000
Liver function	5 (15.6%)	13 (34.2%)	0.076
Thyroid function	1 (3.1%)	8 (21.1%)	0.061
Pre-transfusion check	0 (0.0%)	5 (13.2%)	0.058
Cerebrospinal fluid pressure	1 (3.1%)	6 (15.8%)	0.174
Cerebrospinal fluid protein	4 (12.5%)	18 (47.4%)	0.004^*^
Cerebrospinal fluid virus antibody	0 (0.0%)	16 (43.1%)	<0.001^*^
Cerebrospinal fluid cells	0 (0.0%)	13 (34.2%)	<0.001^*^
Autoimmune encephalitis–related antibody	0 (0.0%)	12 (31.8%)	<0.001^*^
EEG	5 (15.6%)	26 (68.4%)	<0.001^*^
ECG	6 (18.8%)	7 (18.4%)	0.972
Head CT	0 (0.0%)	5 (13.2%)	0.058
Head MRI	0 (0.0%)	24 (63.2%)	<0.001^*^

*EEG, electroencephalography; ECG, electrocardiogram; PCT, procalcitonin; WBC, white blood cell count; DN, department of neurology; DP, department of psychiatry*.

*The data are the results of auxiliary examinations performed in the corresponding departments after the patients were admitted to the hospital*.

# *P-values were obtained with the χ^2^ test for categorical variables*.

* *Significance threshold P < 0.05*.

### Neuropsychiatric Inventory

Overall, the most common neuropsychiatric symptoms among the 70 patients included abnormal behavior (85.7%), delusions (58.6%), hallucinations (52.8%), sleep disturbances (45.7%), and agitation/aggression (44.3%). Specifically, abnormal behavior (97.4%), sleep disturbance (68.4%), delusions (68.4%), hallucinations (60.5%), appetite change (50.0%), and agitation/aggression (42.1%) were most frequently in patients with neurological diseases, whereas for the patients with psychiatric disorders, abnormal behavior (71.9%), irritability (46.9%), delusions (46.8%), agitation/aggression (46.8%), and hallucinations (43.7%) were the most common. There were statistical differences between the two groups, in irritability, abnormal behavior, changes in appetite, and sleep disturbances (*P* < 0.05) (Table 3).

**Table 3:** Neuropsychiatric Inventory (NPI) results.

**Clinical features**	**Two types of diseases (total 70)**		**Admitted to DN (*****n*** **=** **32)**		**Admitted to DP (*****n*** **=** **38)**		***P*-value**^‡^
	**Number**	**%**	**Number**	**%**	**Number**	**%**	
Delusions	41	58.6	15	46.8	26	68.4	0.068
Hallucinations	37	52.8	14	43.7	23	60.5	0.161
Agitation/aggression	31	44.3	15	46.8	16	42.1	0.689
Restlessness/depression	27	38.5	12	37.5	15	39.5	0.866
Anxiety	6	8.6	4	12.5	2	5.3	0.516
Euphoric/high mood	17	24.3	9	28.1	8	21.1	0.492
Indifferent	7	10.0	4	12.5	3	7.9	0.810
Disinhibition	15	21.4	5	15.6	10	26.3	0.278
Irritability	18	25.7	15	46.9	3	7.9	0.001^*^
Abnormal behavior	60	85.7	23	71.9	37	97.4	0.007^*^
Nocturnal behavior	20	28.6	9	28.1	11	28.9	0.940
Changes in appetite	26	37.1	7	21.9	19	50.0	0.015^*^
Sleep disturbances	32	45.7	6	18.9	26	68.4	<0.001^*^

*DN, department of neurology; DP, department of psychiatry*.

*The NPI data were obtained by clinicians in the emergency room*.

‡ *P-values were obtained with the χ^2^ test for categorical variables*.

* *Significance threshold P < 0.05*.

### Comparison of Anti-NMDAR Encephalitis and Schizophrenia

As schizophrenia and anti-NMDAR encephalitis were the most common diseases in the two groups, their clinical features were compared. It was found that the clinical manifestations assessed by Neuropsychiatric Inventory (NPI), laboratory test, and imaging results (Table 4) had statistically significant differences (*P* < 0.05). These differences mainly refer to the number of consultations, history of previous physical diseases, abnormal behavior, sleep disturbances, Babinski sign, cerebrospinal fluid virus antibody, autoimmune encephalitis–related antibody, EEG, and head MRI.

**Table 4:** Results of univariate analysis of anti-NMDAR encephalitis and schizophrenia.

**Variables**	**Anti-NMDAR encephalitis (*n* = 12)**	**Schizophrenia(*n* = 17)**	***P*-value^&^**
Number of consultations (times)	2.7 ± 1.5	1.4 ± 0.7	0.007^*^
History of previous physical diseases	6 (50.0%)	2 (11.8%)	0.038^*^
Abnormal behavior	12 (100.0%)	10 (58.8%)	0.023^*^
Sleep disturbances	10 (83.3%)	1 (5.8%)	<0.001^*^
Babinski sign	5 (41.7%)	0 (0.0%)	0.007^*^
Cerebrospinal fluid virus antibody	5 (41.7%)	0 (17.6%)	0.007^*^
Autoimmune encephalitis–related antibody	12 (100.0%)	0 (0.0%)	<0.001^*^
EEG	12 (100.0%)	3 (17.6%)	<0.001^*^
Head MRI	7 (58.3%)	0 (0.0%)	0.001^*^

*The table shows only the data with statistically significant differences in clinical characteristics, laboratory examinations, imaging, and NPI*.

& *P-values were obtained with the χ^2^ test for categorical variables and the Mann–Whitney U test for continuous variables*.

* *Significance threshold P <0.05*.

## Discussion

The most prominent feature of our study was that we were the first to cross-analyze the clinical characteristics of inpatients from the department of neurology or psychiatry with acute psychiatric symptoms. These patients were first triaged by the emergency nurse and initially misdiagnosed by psychiatrists or neurologists. They had a transfer after rediagnosis. We hope to identify the characteristics of patients with acute psychiatric symptoms in order to achieve early and correct triage in the emergency room.

Our study found that patients diagnosed with neurological diseases are more likely to have somatic symptoms such as dizziness and headaches. On the one hand, this may be caused by the central nervous system disease itself, such as intracranial hypertension, brain stem disease, and prefrontal and temporal Lobe injuries. On the other hand, neurological diseases are often accompanied by somatic symptoms. Research involving 300 patients showed that headaches accounted for the largest proportion of outpatient neurology visits. One hundred forty of them met the diagnostic criteria for mood disorders (*Diagnostic and Statistical Manual of Mental Disorders, Fourth Edition*). Further, the somatic symptoms of patients with mood disorders are more serious than those of patients without mood disorders, which may also be the cause of misdiagnosis (12).

It is worth mentioning that previous studies have suggested abnormal vital signs (e.g., fever) and focal neurological signs (i.e., meningeal irritation and Babinski sign) as red flags of organic diseases (6–8). However, our study found this was not always the case, in the setting of the emergency room. Fever and meningeal irritation may be more indicative of psychiatric disorders, and the Babinski sign was not statistically associated. In our study, 13 patients (40.6%) with fever were mistakenly admitted to the department of neurology. It turned out that the fever was not associated with any neurological diseases, rather, with mental stress, concomitant flu, or infections in other parts of the body. More interestingly, 30 patients (78.9%) who were admitted to the psychiatric department by mistake had a fever within a few days of admission (*P* < 0.05), although their temperature was normal at the time of admission. The same situation applied to the focal nervous system signs, meningeal irritation signs, and Babinski signs. In summary, in the emergency room, all vital signs and focal neurological signs should be recorded immediately, because of the atypical early clinical manifestations. Fever does not necessarily represent the diagnosis of neurological diseases. On the contrary, the absence of fever does not mean they are psychiatric illness. Doctors must be very vigilant to screen carefully.

According to the analysis of the NPI scale, many psychiatric symptoms in these two categories of diseases were overlapping and often atypical. The literature showed that the early clinical manifestations of anti-NMDAR encephalitis and schizophrenia had lots of similarities. Approximately 95% of patients with anti-NMDAR encephalitis have psychiatric abnormal behavior in the early stage, and approximately 75% of them are first treated by a psychiatrist (13–16). Our study revealed that prominent irritability indicated psychiatric diseases (*P* < 0.05), whereas obvious abnormal behaviors, changes in appetite, and sleep disturbances were more suggestive of neurological diseases (*P* < 0.05). When separately analyzing the psychiatric symptoms of patients with anti-NMDAR encephalitis and schizophrenia, we found that 11 of the 12 patients with anti-NMDAR encephalitis (91.7%) had sleep disturbances, whereas there was only one case (5.8%) with schizophrenia (*P* < 0.05). Therefore, sleep disturbance may be an important early symptom of encephalitis, which can provide an important clue for correct and rapid diagnosis, especially in the setting of an emergency room. Previous studies have shown that visual hallucination is a red flag for organic diseases (3). In our study, unfortunately, hallucination had no obvious role in distinguishing these two diseases. When collecting medical history in the future, it may be helpful to further subdivide hallucinations into visual, auditory, and olfactory hallucinations.

After admission, the lumbar puncture, MRI of the head, and EEG will be performed for further diagnosis. If the early clinical symptoms are atypical, or some patients have severe agitation/aggressive behaviors, those examinations usually cannot be completed. However, the abnormality of these examinations is of great significance for the diagnosis of neurological and psychiatric diseases, for example, anti-NMDAR encephalitis and schizophrenia. These abnormalities include detection of cerebrospinal fluid virus antibody, autoimmune encephalitis–related antibody, lesions on head MRI, and characteristic EEG findings. Sometimes, doctors will use antipsychotics to control symptoms or perform diagnostic treatments, but studies have shown that long-term or high-dose use of antipsychotics may induce abnormal EEG and even seizures, which further interferes with the diagnosis (17–19).

## Limitations

There are some shortcomings in this study. (1) The sample size is relatively small. But we have screened all the inpatients with acute psychiatric symptoms from the emergency room for 7 years and cross-analyzed them. Hopefully, in future research, we will combine data from other hospitals to expand the sample size. (2) Only NPI was used to assess the psychiatric symptoms in our study. The symptoms may be better classified and refined if other positive and negative symptoms scales were added. (3) The cognitive functions were not evaluated.

## Conclusion

Acute psychiatric symptoms in the emergency room are usually complex and diverse. Diagnosis can be very challenging. By cross-analyzing inpatient information that misdiagnosed, rediagnosed, and retransferred, clinical characteristics were identified to aid in rapid differential diagnosis promptly.

## Data Availability Statement

The raw data supporting the conclusions of this article will be made available by the authors, without undue reservation.

## Ethics Statement

This study was a retrospective chart review study, ethical review and approval was not required for the study on human participants in accordance with the local legislation and institutional requirements. Written informed consent from the patients was not required to participate in this study in accordance with the national legislation and the institutional requirements.

## Author Contributions

FL, JC, and JM performed material preparation, data collection and analysis. FL wrote the first draft of the manuscript. All authors contributed to the study conception and design, commented on previous versions of the manuscript, and read and approved the final manuscript.

## Conflict of Interest

The authors declare that the research was conducted in the absence of any commercial or financial relationships that could be construed as a potential conflict of interest.

## Abbreviations

NPI: Neuropsychiatric Inventory
EEG: Electroencephalography
ECG: Electrocardiogram
Anti-NMDAR encephalitis: Anti–*N*-methyl-d-aspartate receptor encephalitis
MS: Multiple sclerosis
CJD: Creutzfeldt-Jakob disease
PCT: Procalcitonin
WBC: White blood cell count
DP: Department of Neurology
DN: Department of Psychiatry.
